# The effects of dexamethasone administered during pregnancy on the postpartum spiny mouse ovary

**DOI:** 10.1371/journal.pone.0183528

**Published:** 2017-08-21

**Authors:** Monika Hułas-Stasiak, Piotr Dobrowolski, Bożena Pawlikowska-Pawlęga, Ewa Tomaszewska, Siemowit Muszyński

**Affiliations:** 1 Department of Comparative Anatomy and Anthropology, Maria Curie-Sklodowska University, Lublin, Poland; 2 Department of Animal Physiology, Faculty of Veterinary Medicine, University of Life Sciences, Lublin, Poland; 3 Department of Physics, Faculty of Production Engineering, University of Life Sciences, Lublin, Poland; INIA, SPAIN

## Abstract

Excessive exposure to glucocorticoids can alter ovarian function by modulating oogenesis, folliculogenesis and steroidogenesis. The aim of the present study was to examine the effects of dexamethasone (DEX) administered during pregnancy on folliculogenesis and corpus luteum development in the postpartum spiny mouse ovary. DEX (125 μg kg^-1^ body weight per day) was applied to pregnant spiny mouse from day 20 of gestation to parturition. The obtained ovaries were fixed and used for immunohistochemistry and TEM analysis. The expression of proteins related to apoptosis (caspase-3, Bax, Bcl-2) and autophagy (Beclin1, Lamp1) as well as PCNA and GR receptors were evaluated by western-blot. In comparison with DEX-treated group a higher percentage of TUNEL positive granulosa and luteal cells was observed in the control group. These data were consistent with changes in caspase-3 and Bax expression, which increased in the control and decreased after DEX exposure. In turn, the proliferation index and PCNA expression were higher in the DEX-treated group. Moreover, the higher level of Beclin1, Lamp1, anti-apoptotic Bcl-2 protein and GR was observed in the DEX-treated females than in the control group. Beclin1 and Lamp1 were strongly expressed in luteal cells which exhibited an autophagic ultrastructure. Surprisingly, DEX augmented the number of ovarian follicles and corpora lutea, which resulted in a significant increase in ovarian weight. These findings suggest that DEX exerts anti-apoptotic action on granulosa layer and stimulates follicular maturation. Moreover, DEX induces autophagy in luteal cells promoting cell survival rather than cell death, which can prolong the corpus luteum life span.

## Introduction

Excessive exposure to glucocorticoids (GC) can affect ovarian function by modulating oogenesis, folliculogenesis, and steroidogenesis [1, 2]. They exert their biological effects directly through glucocorticoid receptors (GR) located in ovarian cells and indirectly through the hypothalamus-pituitary axis [2].

An appropriate balance between cell death and proliferation may determine whether a follicle will continue to develop or undergo atresia as well as it is essential for corpus luteum (CL) formation or regression [3–5]. Uncontrolled atresia (caused by environmental toxicants as well as by lifestyle) mediated by programmed cell death (apoptosis and/or autophagy) would significantly reduce the number of available ovarian follicles [6, 7]. Similarly, enhanced luteolysis, especially during pregnancy, can diminish luteal progesterone production and lead to abortion of the foetuses, particularly in these species in which CL is necessary for the continuation of normal pregnancy [8].

During apoptosis, active caspase-3 plays the central role. This enzyme is responsible for the proteolytic cleavage of cellular and nuclear substrates that leads to typical morphological changes of apoptosis [9, 10]. Apoptosis is also accompanied by changes in Bax (pro-apoptotic factor bcl-2-associated X protein) and Bcl-2 (anti-apoptotic factor B cell lymphoma/leukemia2) expression [11]. Furthermore, autophagy has been proposed as an important non-apoptotic mode of cell destruction [12, 13].

Autophagy is an intracellular bulk degradation system in which a portion of the cytoplasm is enveloped in double membrane–bound structures called autophagosomes which undergo maturation and fusion with lysosomes to form autolysosomes [14]. Then, the sequestered intra-autophagosomal components are degraded by lysosomal hydrolases. Autophagy is a complex process involving many proteins. One of them is Beclin 1, which is important for localization of autophagic proteins necessary to form a pre-autophagosomal structure (phagophore). Moreover, Beclin 1 can be involved in autophagosome/endosome maturation. Many of these effects are mediated through the autophagic inducers and inhibitors [15]. Beclin1 also directly interacts with the anti-apoptotic protein, Bcl-2. Therefore, it is recognized as a seminal protein in bridging autophagy and apoptosis [16–18]. The other proteins involved in autophagy are the lysosomal transmembrane glycoproteins Lamp1 and Lamp2. They are necessary for lysosomal fusion with endosomes, autophagosomes, and cell membrane [19].

The previous study indicated that prenatally administered synthetic glucocorticoid—dexamethasone (at a dose 125 μg/kg/bw) caused ovarian toxicity in the spiny mouse offspring [7]. DEX enhanced follicular atresia by activating programmed cell death, which in turn depleted the stock of ovarian follicles. On the other hand, DEX stimulated the development of the granulosa-cell layer and primary to secondary follicle transition. However, the prolonged action of DEX induced atretic changes in mature secondary follicles, which were predominantly visible in the oocyte.

Therefore, the aim of the present study was to examine whether DEX affected folliculogenesis and CL function in the maternal spiny mouse ovary during pregnancy. Thus, 1) the pattern of ovarian activity (proliferation index), 2) the effect of DEX on apoptosis and autophagy in ovarian follicles and the CL, and 3) the pattern of GR localization and expression in the spiny mouse postpartum ovary were examined.

The spiny mouse *(Acomys cahirinus*) was used as an experimental model for several reasons. It is a rodent species with a relatively long period of pregnancy (39–40 days) and a few offspring (1–5).The spiny mouse is a precocial species characterized by rapid development of the foetus in which organogenesis is largely completed by the end of gestation. The young are furred, have open eyes and they are able to move just after birth. The spiny mouse indicates an advanced stage of ovary maturation at the time of birth which is the result of folliculogenesis initiated in foetal life [20]. In contrast, in laboratory rats and mice follicular development starts during postnatal period. This makes the spiny mouse an ideal animal model for foetal and neonatal studies. *Acomys cahirinus* is an unusual rodent with organogenesis at birth closely resembling that of the human infant, much more than any other known rodent species. Moreover, unlike common rodents but similar to the human, the foetal adrenal gland of the spiny mouse can synthesize dehydroepiandrosterone and cortisol [21]. Additionally, the spiny mouse is the first rodent species known to undergo spontaneous decidualisation and menstruation. It provides an unprecedented non-primate model to study the mechanism of menstrual shedding and repair, and may be useful in understanding human specific menstrual and pregnancy associated diseases [22].

## Materials and methods

The experiment was approved by The Local Ethics Committee on Animal Experimentation of Maria Curie-Skłodowska University, Lublin, Poland (2013/55).

### Experimental design

The spiny mice *(Acomys cahirinus)* used in this experiment were obtained from our own laboratory colony. Randomly assigned dams were divided into control (n = 14) and DEX-treated groups (n = 14) based on body weight (40–50 g). Experimental females were mated with males in a 1:1 ratio and allowed to naturally deliver. The spiny mouse exhibited a postpartum oestrus within 24 hours after delivery, therefore this period was used as the day of conception and the next day as the first gestation day.

Pregnant dams were maintained separately in standard rodent cages at 22°C and 55–60% humidity on a 12L:12D photoperiod. The DEX-treated group received dexamethasone (Dexamethasone, Tab. 0.5 mg, Polfa, Pabianice, Poland) *per os* at a dose of 125 μg/kg/per from day 20 of gestation until parturition. The tablets were crushed and incorporated within the feed pellets at an appropriate concentration, adjusted to the body weight of animals. The chosen dose was based on data published by Dickinson group [23]; however, the route and time of DEX administration were modified.

The animals were fed twice daily (at 8 am and at 12 pm) with a standard diet (Agropol S.J., Poland) and had free access to fresh water. At the begining of the experiment the total amount of food consumed by the dams was calculated as three pellets (about 15 g) feeding stuff per day. In the morning, the DEX-treated group received one pellet with DEX and the control females received one mechanically changed pellet but without DEX. There was no vehicle administration to the control group except mechanical manipulation of one pellet to achieve the texture similar as the mixed with dexamethasone in the DEX group. At 12 pm, the consumption of pellet was checked and the remaining two pellets were given to the both groups. There were no differences in food and water intake between the groups. The gestation length (39–40 days) and the number of litter size (2 to 4) did not differ between both groups examined. Within the first 24 h after parturition, dams from the control and the DEX-treated group were euthanized by CO_2_ inhalation.

### Tissue preparation

Ovaries from 6 adult females per group (24 ovaries in total) were excised, fixed in phosphate buffered 4% paraformaldehyde, dehydrated, and embedded in Paraplast (Sigma-Aldrich, USA). The 5 μm-thick ovarian sections were cut on a rotary microtome and placed on polysine-coated glass slides (SuperFrost Plus, Germany), numbered, and processed for routine hematoxylin-eosin (H&E) staining and immunohistochemistry.

### Follicle counts

The total number of ovarian follicles was counted as described by Tilly [24]. Three randomly selected slides (seven ovarian sections per slide) from each ovary (n = 12 per group) were evaluated quantitatively. Every 3rd section was chosen to analysis. The number of primordial and primary follicles calculated was then multiplied by a corrector factor of 15 (x 3-remainderm, unanalysed sections; and x 5-section thickness) to estimate the total number of follicles per ovary. More advanced follicles (secondary type and beyond) and corpora lutea were estimated by exact counts of H&E stained sections encompassing whole cross-sections of the ovary. Follicular counts were made using a x 40 objective on an Eclipse E-800 light microscope. The oocyte nuclei number was equated to the follicular number. Follicles were identified according to the Pedersen and Peters classification system [25]. Primordial follicles were defined as an oocyte surrounded by a partial layer of squamous granulosa cells, primary follicles possessed single layer of cuboidal granulosa cells, follicles with more than one layer of granulosa cells without visible antrum were classified as secondary follicles, antral follicles with the antral space between the granulosa cell layer, and preovulatory follicles with a rim of cumulus cells surrounding the oocyte. Atretic follicles were classified on the basis of structural alterations observed in oocytes (cytoplasmic vacuolization) as well as in the granulosa layer (disintegration, apoptotic bodies).

### Immunohistochemistry

Paraplast sections were dewaxed in xylene, rehydrated in ethanol and rinsed in water. Next the sections were heated in a microwave oven (3x5 min in 10 mM citrate buffer, pH 6.0) to retrieve antigenicity. Endogenous peroxidase activity was blocked by incubation with 3% H_2_O_2_ in methanol (1:1) and nonspecific binding was prevented with 5% goat serum (Sigma-Aldrich, USA). Then the following primary antibodies: anti-caspase-3 (1:50, Sigma-Aldrich), anti-Lamp1 (1:50, Santa Cruz Biotechnology, Santa Cruz, CA) anti-Beclin1 (1:50, Santa Cruz Biotechnology, Santa Cruz, CA), anti-PCNA (mouse monoclonal antibody 1:50, Santa Cruz Biotechnology, Santa Cruz, CA) and anti- GR (1:50, Santa Cruz Biotechnology, Santa Cruz, CA) were used to tissue sections and incubated overnight at 4°C. The antigens were visualized using corresponding biotinylated secondary goat anti-rabbit or anti-mouse antibodies (1:200, 1 h at room temperature (RT), Abcam, Cambridge, UK), avidin-biotin-peroxidase complex (1:300, 30 min at RT; StreptABCcomplex-HRP, Dako, Glostrup, Denmark), and 3,3’-diaminobenzidine (DAB, Sigma-Aldrich) as a chromogen staining substrate. The sections were rinsed with Tris-buffered saline (TBS, pH 7.6) after each step of the above procedure. Next, the slides were counterstained with Mayer’s hematoxylin, dehydrated and mounted in DPX. Finally, the selected slides were analysed under a Nikon Eclipse-800 microscope with a Nikon D200-digital camera (Nikon, Tokyo, Japan). The negative controls were performed either in the absence of primary antibody or with the use of non-immune IgG. Proliferation index (PI) was calculated by dividing the number of PCNA-positive granulosa or luteal cells of ovarian follicles or corpora lutea by the total number of cells in the relative compartments and the result was multiplied by 100. At least 10 different sections from each animal were examined. The data were expressed as mean ± s.e.m.

### Terminal deoxynucleotidyl transferase-mediated dUTP Nick end-labelling (TUNEL) staining reaction

The paraplast sections were mounted on poly-lysine-coated glass slides. After deparaffinisation and rehydration, the slides were heated in a microwave oven (3 min in 10 mM citrate buffer, pH 6.0), rinsed in PBS. The in situ cell death detection kit, POD (Roche Diagnostics, Switzerland) was used and the protocol of the, POD (Roche Diagnostics, Switzerland)manufacturer was followed. All the sections were counterstained with Mayer’s hematoxylin and examined under a light microscope (Nikon Eclipse-800). Apoptotic index (AI) was calculated by dividing the number of TUNEL-positive granulosa or luteal cells of ovarian follicles or corpora lutea by the total number of cells in the relative compartments and the result was multiplied by 100. At least 10 different sections from each animal were examined. The TUNEL assay was repeated two times for each sample with similar results. The data were expressed as mean ± s.e.m.

### Transmission electron microscopy (TEM)

Isolated ovaries (n = 4, from 2 females per group) were fixed in 4% glutaraldehyde in sodium cacodylate buffer (0,1 M, pH 7.4) for 2 h and postfixed with 1% osmium tetroxide in the same buffer for 2h. After dehydration in a graded ethanol series, the specimens were placed in propylene oxide and embedded in LRWhite. Ultrathin sections were contrasted with uranyl acetate and lean citrate, and examined by electron microscopy LEO 912 AB (Zeiss, Germany).

### Western-blot analysis

The ovaries obtained from the DEX-treated and the control females (n = 6 per group) were homogenized in lysis buffer (125 mM TRIS-HCl pH 6.8; 4% SDS; 10% glycerol; 100 mM DTT), boiled in water bath for 10 min, and centrifuged at 10000 x g for 10 min, and the supernatant was collected. The protein concentration was determined by the Bradford method [26] and samples of supernatants containing 80μg of proteins were separated on 10% SDS-PAGE and subsequently electroblotted onto an Immobilon P membrane (Sigma-Aldrich). After blocking with 3% low fat milk in PBS for 1h, the membranes were incubated overnight with the following primary rabbit antibodies: anti-caspase-3 (1:1000), anti-Bcl-2 (1:1000, Santa Cruz Biotechnology, Santa Cruz, CA), anti-Bax (1:300, Abcam, Cambridge, UK), anti-Lamp1 (1:300), anti-Beclin1 (1:300), and anti-GR (1:1000) as well as with anti-PCNA mouse monoclonal antibody. Next, the membranes were washed 3x10 min in PBS containing 0.05% TRITON X-100 (Sigma) and incubated with alkaline phosphatase-conjugated goat anti-rabbit or anti-mouse IgG (Sigma-Aldrich, 1:30 000, for 2h).The signals were visualized using BCIP (5-bromo-4-chloro-3indolyl phosphate) and NBT (nitrotetrazolium blue chloride), which gives a blue reaction product. Each membrane was reprobed with anti-actin antibody (1:2000, Sigma-Aldrich, St. Louis, Mo, USA) which was used to control for variable amounts of protein. The analysis of images was performed using the public domain ImageJ program with “Gel Analysis” function. The bands were densitometrically quantified and normalized to their corresponding ß-actin bands. Semi-quantitative analysis was performed for three separately repeated experiments from each control and DEX-treated groups.

### Image analysis

To estimate the intensity of immunohistochemistry reaction (IHC) quantitatively for the following proteins: GR, caspase-3, Lamp1, and Beclin1, digital images from at least 12 sections of ovaries from the each control and DEX-treated females were analysed. In total, 144 sections were subjected to image analysis (12 ovarian sections per 1 animal, n = 6 in each group). The slides were processed immunohistochemically at the same laboratory conditions in order to obtain comparable staining intensities. The digital colour images were registered using confocal microscope LSM 5 Pascal (Carl Zeiss, Jena, Germany) equipped with a camera AxioCam HRc (Carl Zeiss, Jena, Germany) and connected to a computer. Images of primordial, primary, and secondary ovarian follicles were captured by a 40x objective; in turn, antral and preovulatory follicles as well as corpora lutea were registered by a 20x objective. All images were saved as 24 bit RGB colour images in TIFF (Tagged Image File Format) for quantification. A colour threshold function of ImageJ (Wayne Rasband, National Institute of Mental Health, Bethesda, Maryland, USA) was used to separate DAB stained pixels from the background. To define colour threshold range for DAB, the hue, saturation and brightness for the randomly chosen sample of images were established. Then, the selected threshold was applied to extract the positively stained colour pixels (DAB) from the background (hematoxylin stained pixels) in the analysed images. The theca cells, granulosa layer and oocyte from the selected follicles were carefully dissected from each other to avoid contamination of the untargeted cells The results of 10 separate measurements (per sections) for each primordial, primary, and secondary follicle as well as 5 measurements for antral follicles, and 2 or 3 for preovulatory follicles and corpora lutea were expressed as mean ± s.e.m.

The intensity of IHC reaction was evaluated in the 8-bit greyscale. In digital image analysis, the pixel intensity values range from 0 to 255, wherein 0 represents the black and 255- white shade of grey. In order to facilitate illustration of the obtained results (graphs in the text) the pixel brightness values were measured on inverted images in the 8-bit greyscale (from 0-white to 255-black).

### Statistical analysis

All results are expressed as means ± s.e.m, and differences were considered statistically significant at *P*<0.05 The differences between the means were tested with the Student’s t-test (two-tailed test). To verify the normal distribution of data the Shapiro-Wilk test was used. In the case of a lack of normal distribution and/or unequal variance of data Mann-Whitney *U*-test was applied to determine significant differences between the control and the DEX-treated groups. All statistical analyses were performed using STATISTICA 8.0 program (Statsoft Inc., Tulsa, OK, USA).

## Results

### General observations

The signs of discomfort, behavioural anomalies, sickness, or mortality were not observed in the pregnant females during the study period. The DEX treatment had no effect on maternal weight gain on day 39 of pregnancy (DEX: 60.4±7.5 g, Control: 61.5±5.9 g), but significantly increased ovarian weight (DEX: 11.8±4.2 mg; Control: 6.6±3.4 mg, p<0.001).

### Morphological and ultrastructural observations

The histological evaluation of the spiny mouse postpartum ovary revealed the presence of follicles in all stages of development (primordial, primary, secondary, antral, and preovulatory) both in the control and the DEX-treated group. The number of healthy and atretic primordial and primary follicles was similar in the control and the DEX-treated groups (Table 1). However, the mean number of healthy secondary, antral, and preovulatory follicles was significantly greater (p<0.001, p<0.001 and p<0.01 respectively) in the DEX-treated females. The follicles were localized in the outer part of the cortex near the epithelium. The inner part of the cortex was filled with large CL. The diameters of CL in the DEX-treated group were greater (p< 0.001) than in the control (DEX: 944±32 μm; Control: 591±37 μm). Furthermore, small, regressing CLs (250.4±42 μm) which presumably came from the previous pregnancy were present in the control, but not in the DEX-treated group. Moreover, the number of CLs was greater (p<0.001) in the DEX-treated than control group (Table 1).

**Table 1:** Number of total follicle types and corpora lutea in the postpartum spiny mouse ovary.

Follicle type	Control	DEX-treated
Healthy primordial	436.05 ± 6.8	427.2 ± 9.2
Atretic primordial	83.85 ± 2.6	82.05 ± 2.9
Healthy primary	287.55 ± 6.2	275.85 ± 3.6
Atretic primary	71.55 ± 2.5	64.65 ± 2.04
Healthy secondary	3.9 ± 0.3	7.72 ± 0.3***
Atretic secondary	4.66 ± 0.2	2.20 ± 0.2**
Healthy antral	1.52 ± 0.1	4.81 ± 0.1***
Atretic antral	2.46 ± 0.2	1.25 ± 0.09**
Healthy preovulatory	1.17 ± 0.08	2.88 ± 0.1**
Atretic preovulatory	0.8 ± 0.1	0.6 ± 0.2
Corpus luteum (CL)	0.52 ± 0.1	2.85 ± 0.1***
Degenerated CL	1.22 ± 0.8	not observed

Data are presented as mean ± s.e.m. P-values less than 0.05 from Student’s t-test are indicated

**p<0.01.

***p<0.001.

**Fig 1:**
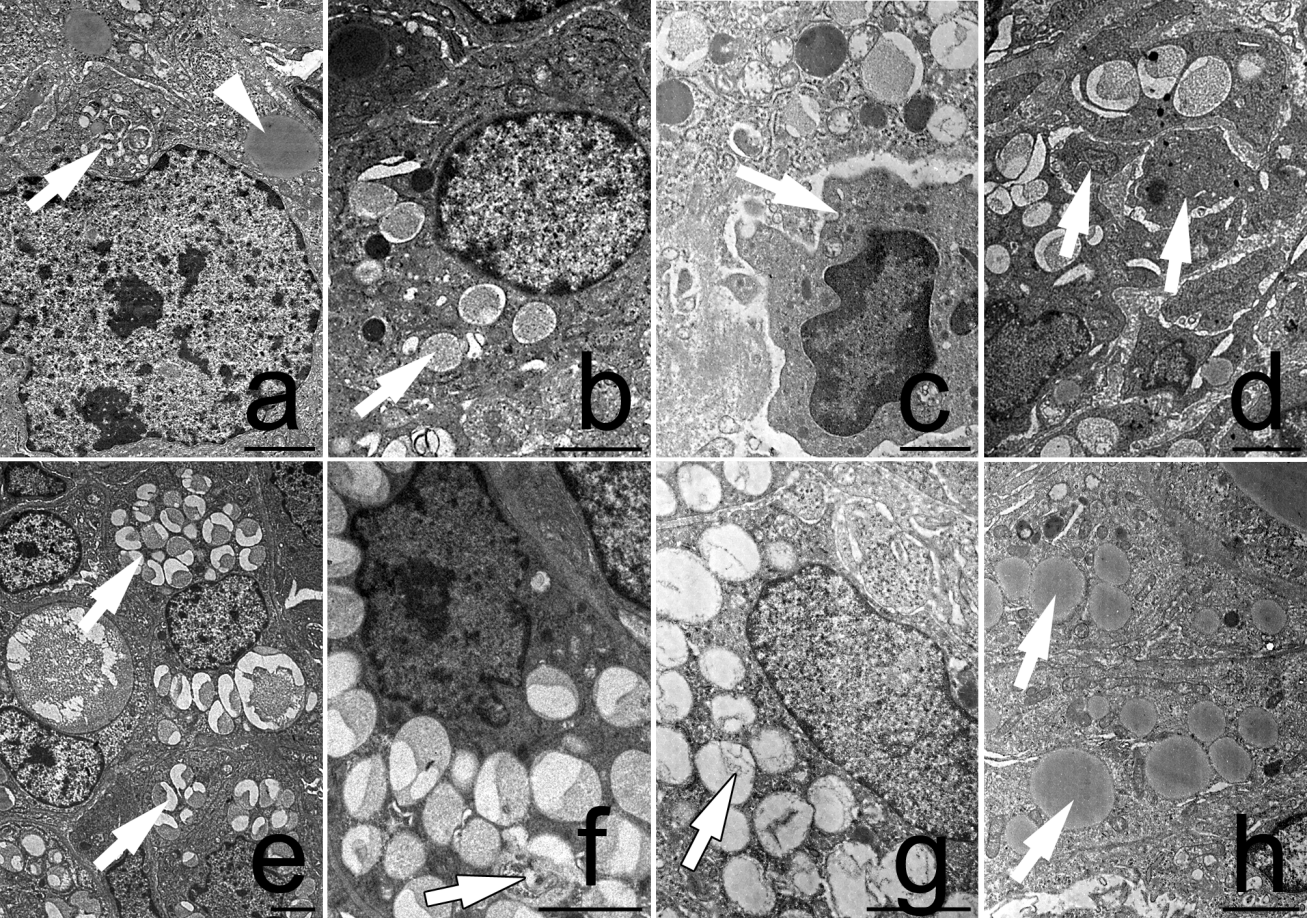
**Ultrastructure of luteal cells from the control (a, b, c, d) and the DEX-treated females (e, f, g, h).** The autophagosome (arrow) and lipid droplets (arrowhead) were visible in the cytoplasm of the control luteal cells (a). Autolysosomes (arrow) were also present within luteal cells (b). The luteal cells from the control group underwent degeneration (c, d). They had a pyknotic nucleus (arrow, c) and became fragmented (arrows, d). The autophagosomes (arrow) were observed in the luteal cells from the DEX-treated females (e). Note a large number of autolysosomes which filled the whole luteal cell (arrow, f, g). The integrity of the cell was maintained. Abundant lipid droplets were visible in the luteal cells from the DEX-treated females (h). All the scale bars represent 2μm.

The ultrastructural analysis of large luteal cells from the DEX-treated group indicated the characteristic features of autophagy: the presence of numerous autophagosomes as well as absence of chromatin condensation (Fig 1E, 1F and 1G). The accumulation of lipid droplets in the cytoplasm was also observed (Fig 1H). Moreover, these luteal cells maintained their integrity and did not exhibit apparent degenerative changes. In turn, the luteal cells from the control group showed combinations of dense granules and autophagic vacuoles in the cytoplasm. However, the amount of autophagic structures in luteal cells from the control group was lower (Fig 1A and 1B) in comparison with the DEX-treated females (Fig 1E and 1F). Finally, the control luteal cells underwent degenerative alterations and became fragmented (Fig 1C and 1D).

### Proliferative activity (PCNA)

To assess cell proliferation, the proliferating cell nuclear antigen protein (PCNA) was used. This protein starts to accumulate in G1 phase of cell cycle, reaches the highest level during the S phase and decreases during G2/M phase. Therefore, PCNA is a useful tool to study cell proliferation [27].

**Fig 2:**
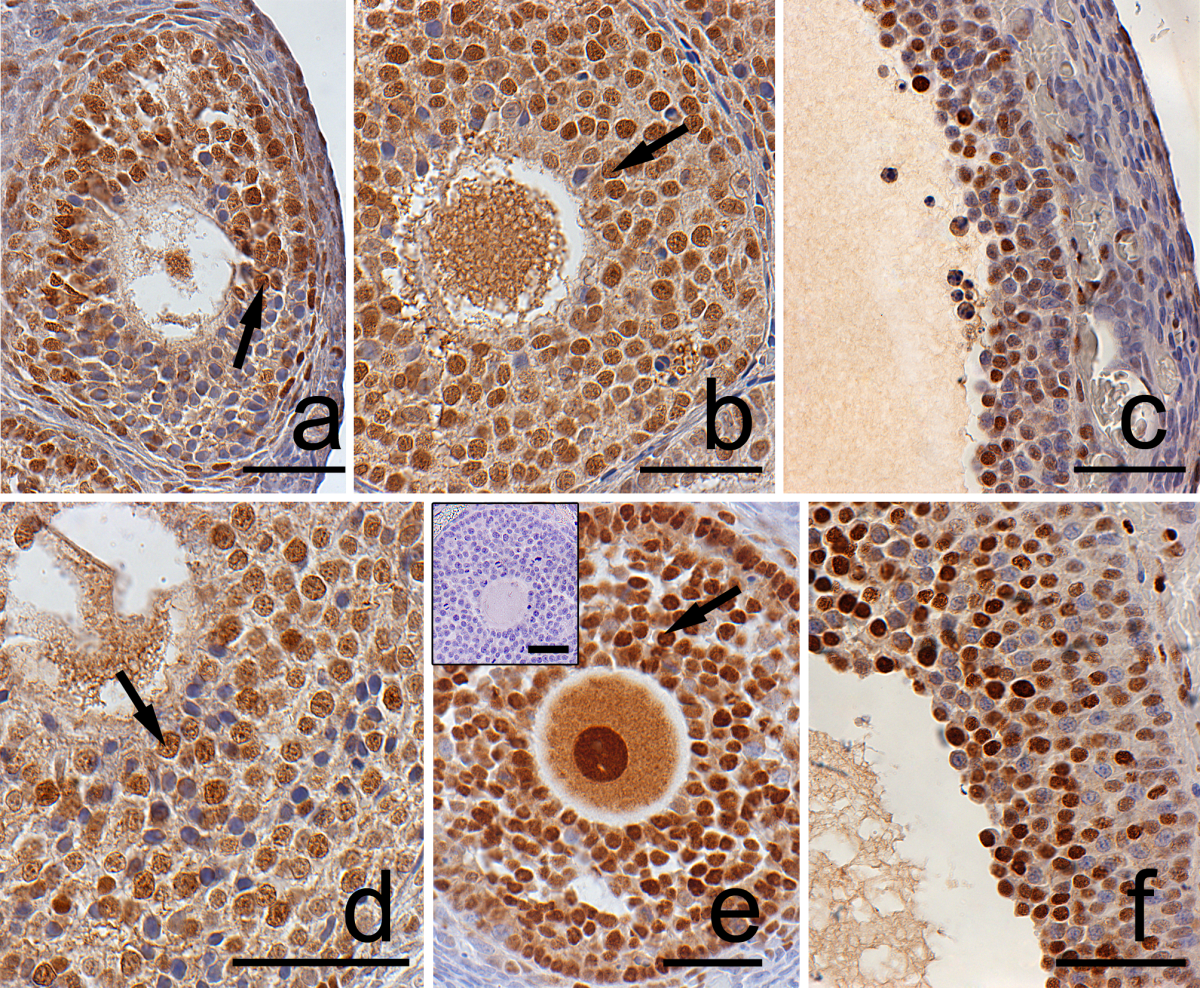
**Localization of PCNA in granulosa layer of ovarian follicles from the control (a, b, c) and DEX-treated females (d, e, f).** The positive PCNA staining (arrows) was detected in granulosa cells of atretic secondary (a) as well as in healthy secondary (b) and preovulatory (c) follicles from the control females. Similarly, in the DEX-treated group PCNA staining was observed in atretic secondary (d), healthy antral (e) and preovulatory follicles (f). The control section did not exhibit any positive staining (e, inset). Scale bars represent 50μm.

**Fig 3:**
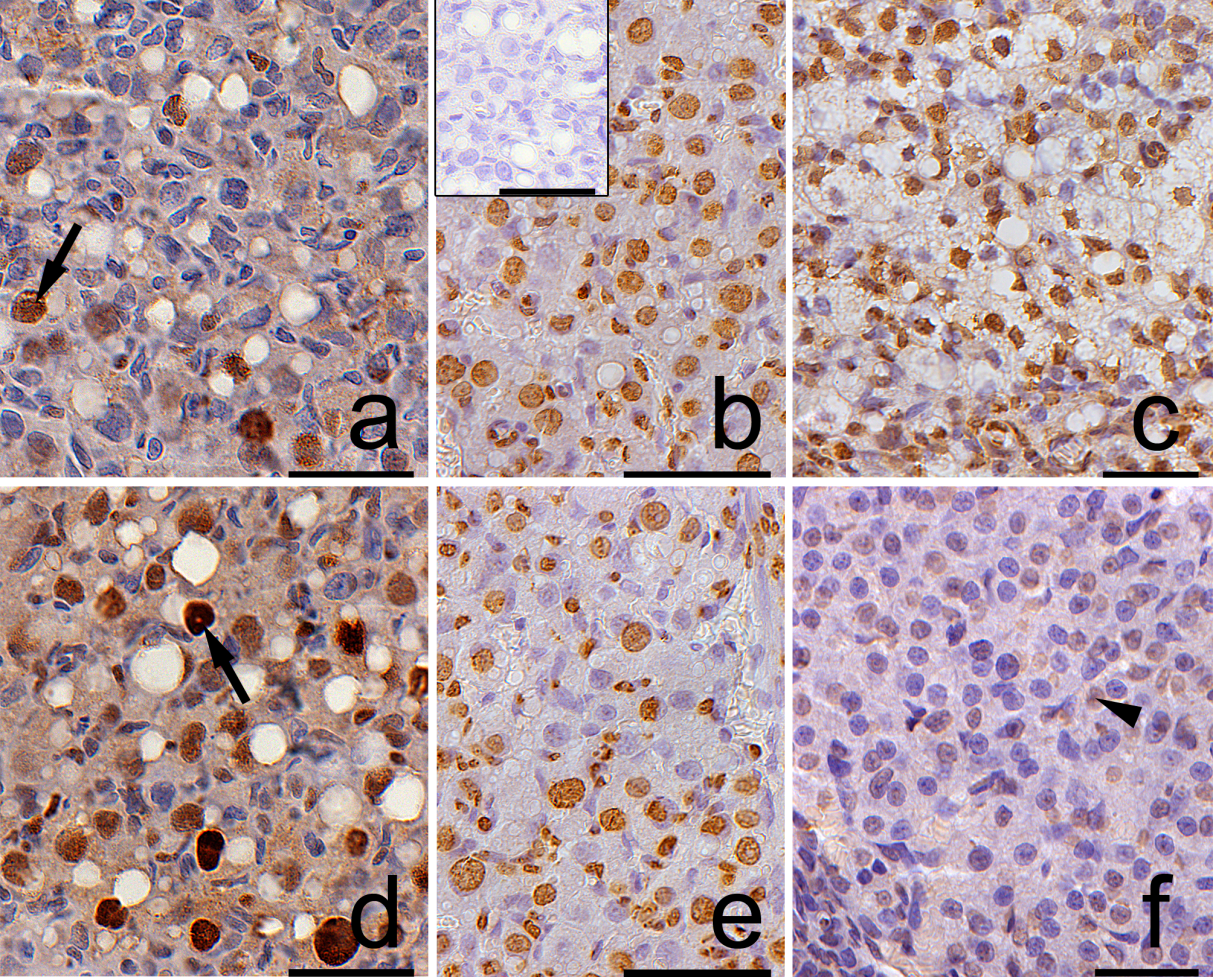
**Immunolocalization of PCNA and TUNEL-positive cells in the postpartum corpora lutea from the control (a, b, c) and the DEX-treated females (d, e, f).** The positive PCNA staining (arrows) was observed both in the control (a) and the DEX-treated group (d). Apoptotic (TUNEL-positive) luteal cells were also visible in the control (b, c) as well as in the DEX-treated group (e). The control luteal cells at the late stage of CL regression were filled with numerous vacuoles which combined into one big vacuole causing cell disintegration. Moreover, most of them were TUNEL-positive (c). The negative control (TUNEL assay) was conducted without the terminal deoxynucleotidyl transferase enzyme (b, inset). Fully functional CL from cycling, non-pregnant spiny mouse was stained for TUNEL and used as a positive control (f). Scale bars represent 50μm.

In the postpartum spiny mouse ovary PCNA was localized in granulosa and theca cells as well as in oocytes of all follicle types both in the control (Fig 2A, 2B and 2C) and the DEX-treated group (Fig 2D, 2E and 2F). Furthermore, large, differentiated luteal cells from the control (Fig 3A) and the DEX-treated group (Fig 3D) were also PCNA-positive. In the DEX-exposed group the percentage of PCNA-immunopositive cell (proliferation index, PI) in granulosa layer of ovarian follicles as well as in CL was significantly higher (p<0.001) when compared to the control (Table 2).

**Table 2:** Effect of dexamethasone on the proliferation index (PI) of granulosa and luteal cells in the postpartum spiny mouse ovary.

Healthy ovarian follicles	Proliferation index (PI) %	
	Control	DEX-treated
primary	60.2 ± 1.3	81.1 ± 2.0 **
secondary	61.3 ± 3.5	82 ± 2.0 **
antral	56.4 ± 1.6	79 ± 2.3 **
preovulatory	42.5 ± 2.3	58.6 ± 1.5 **
luteal cells	39.7 ± 4.8	63.7 ± 2.7 ***

Values are expressed as mean ± s.e.m. P values were calculated with the Mann Whitney U-test

**p<0.01.

***p<0.001.

### Apoptosis and autophagy markers

**Fig 4:**
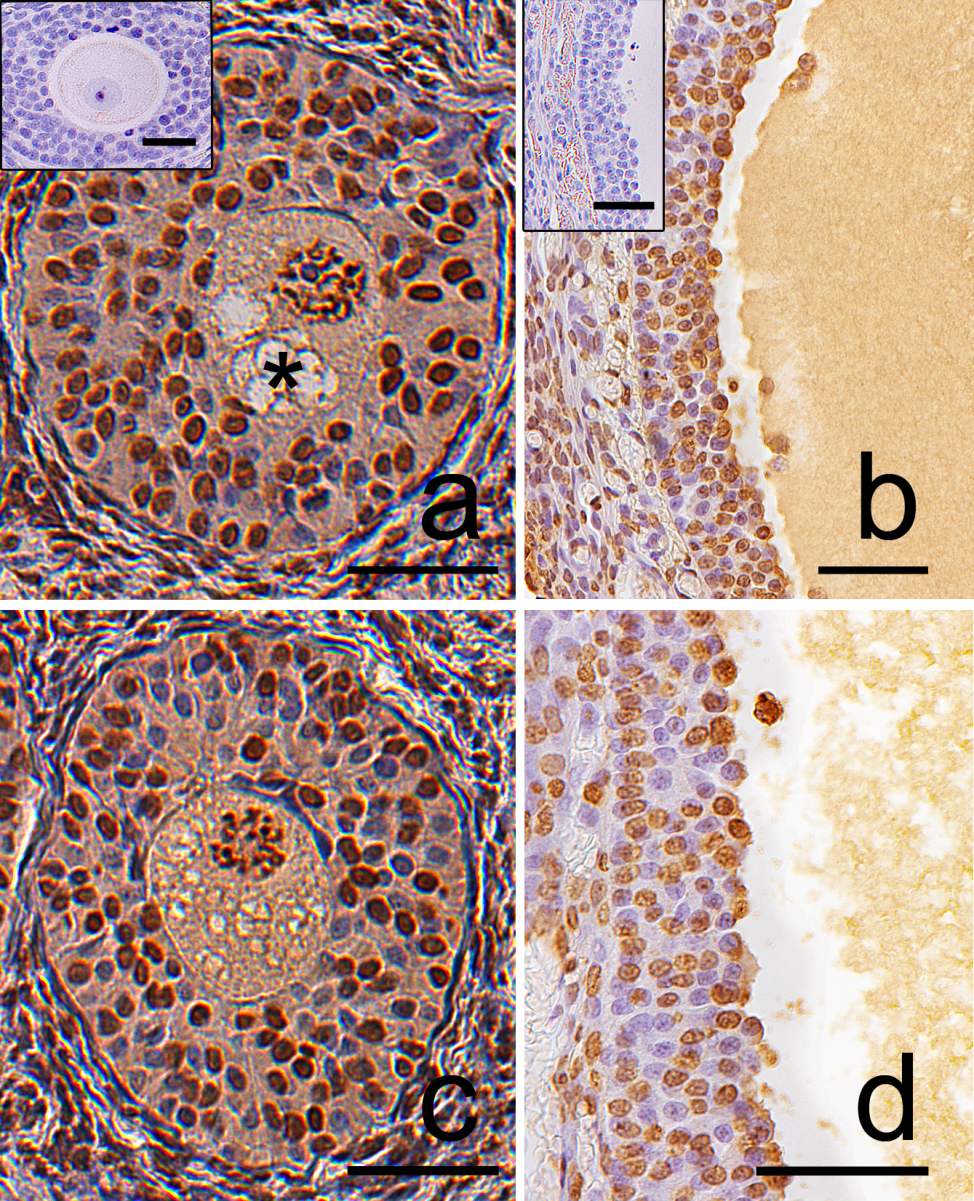
**Localization of apoptotic cells in granulosa layer of ovarian follicles from the control (a, b) and the DEX-treated females (c, d). Apoptotic cells (arrows) were identified using TUNEL method.** The control sections did not exhibit any positive staining (a,b, inset). Scale bars represent 50μm.

**Fig 5:**
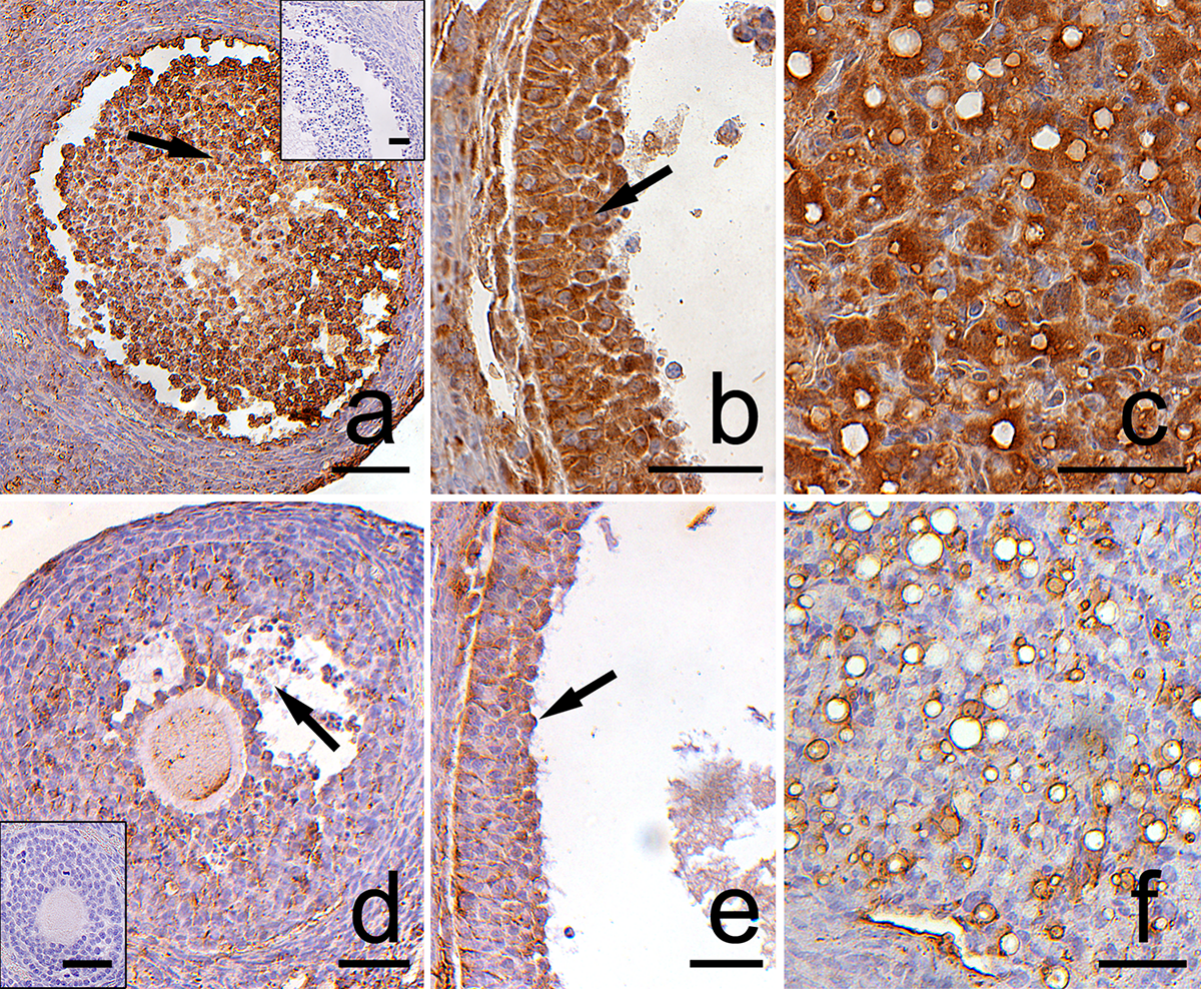
**Immunolocalization of caspase-3 in the postpartum spiny mouse ovary from the control (a, b, c) and the experimental group (d, e, f).** The caspase-3 staining was visible in the granulosa cells (arrows) of atretic ovarian follicles as well as in regressing luteal cells (arrowhead) both in the control (a,b,c) and the DEX-treated females (d,e,f). However, the intensity of caspase-3 was evident in the control group. The control sections did not exhibit any positive staining (a,d, inset). All the scale bars represent 50μm.

**Fig 6:**
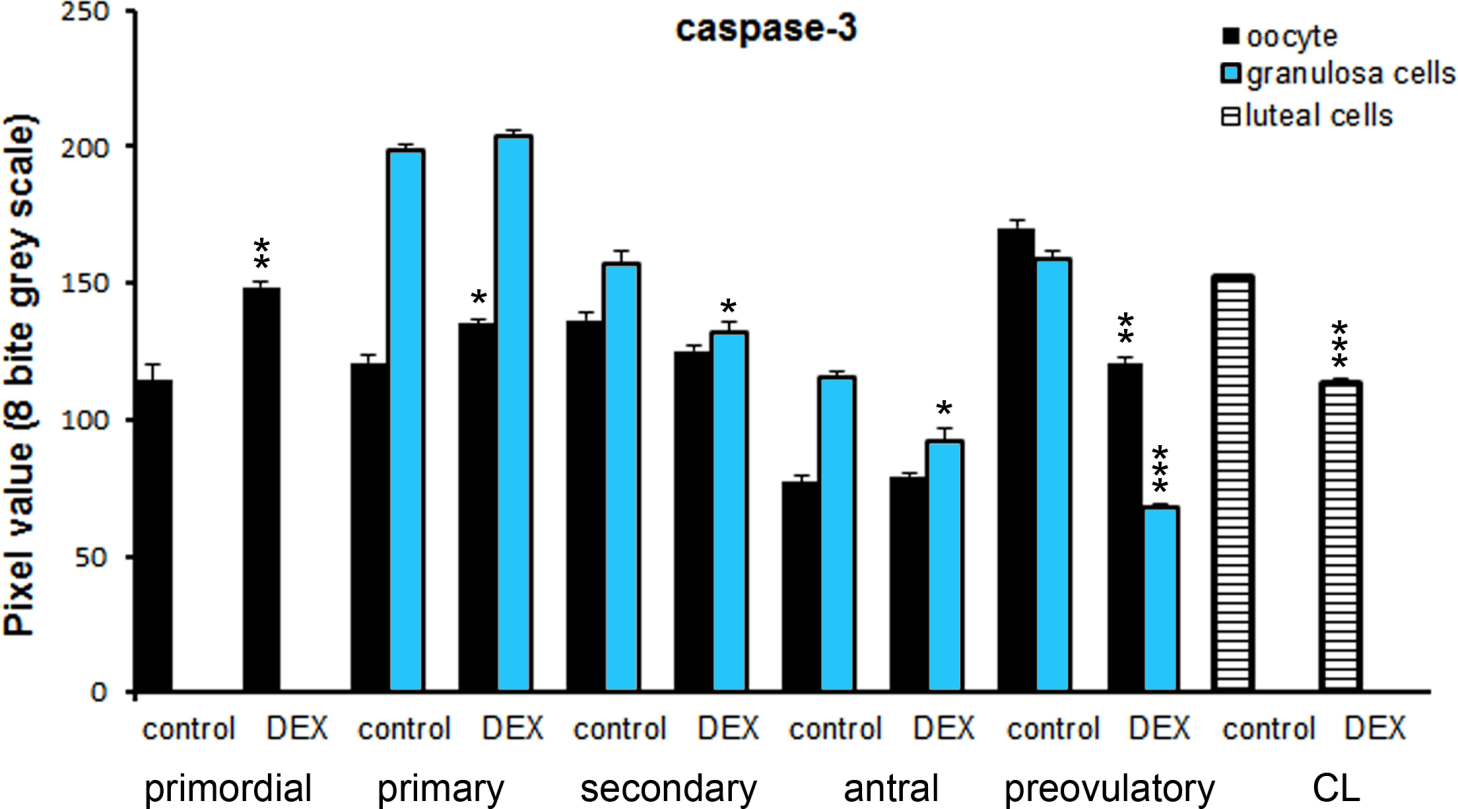
Quantitative analysis of the intensity of caspase 3 staining in ovarian follicles/corpus luteum from the control and the DEX-treated females. Data are presented as mean ± s.e.m. P-values less than 0.05 from Student’s t-test are indicated *p<0.05, **p<0.01, ***p<0.001, CL- corpus luteum.

**Fig 7:**
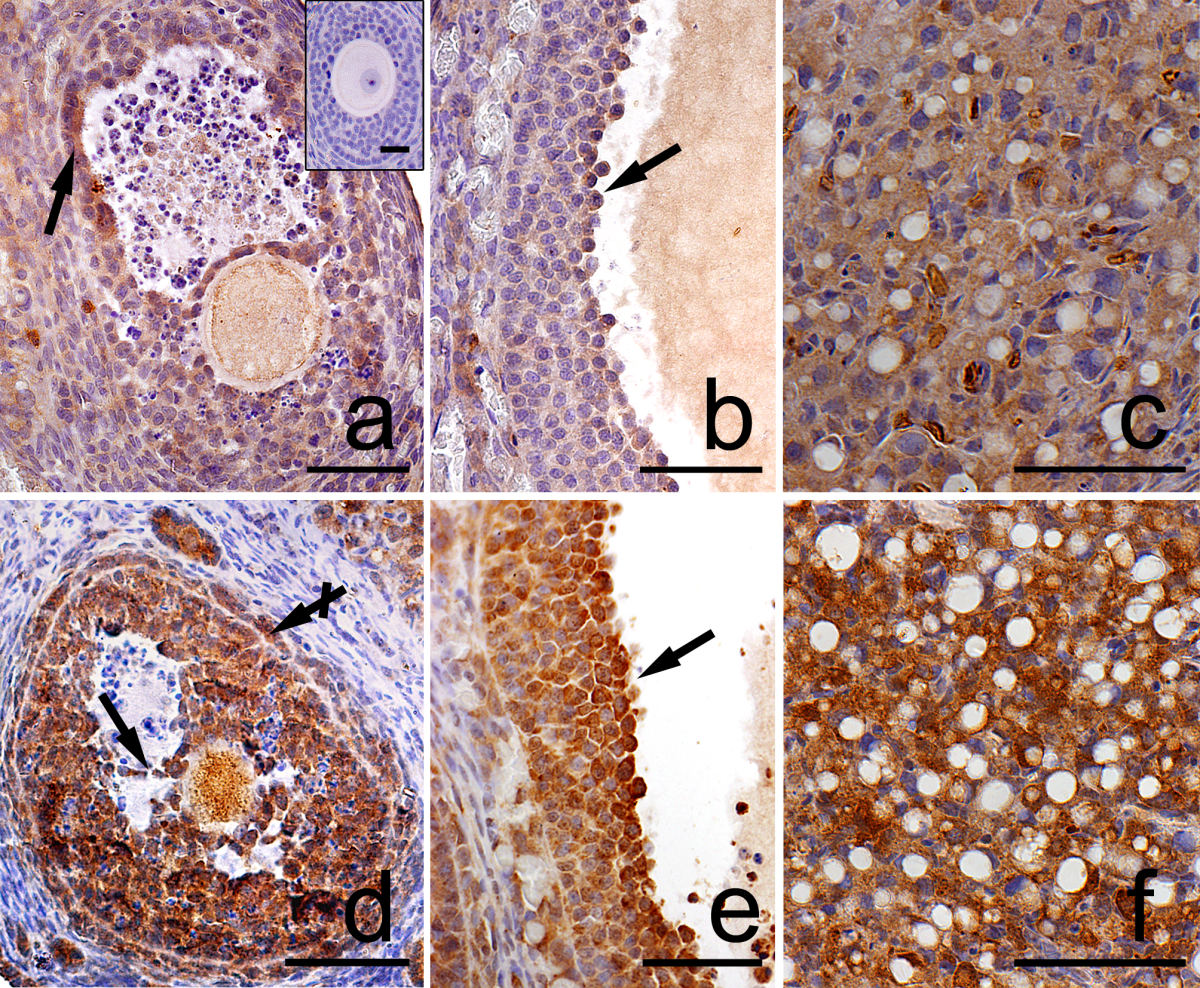
**Immunolocalization of Lamp1 in the postpartum spiny mouse ovary from the control (a, b, c) and the DEX-treated females (d, e, f).** Micrographs showed more intensive Lamp1 staining in granulosa (arrows) and theca (crossed arrow) cells as well as in luteal cells in the DEX-treated group (d,e,f) than in the control (a,b,c). The control section did not exhibit any positive staining (a, inset). All the scale bars represent 50μm.

**Fig 8:**
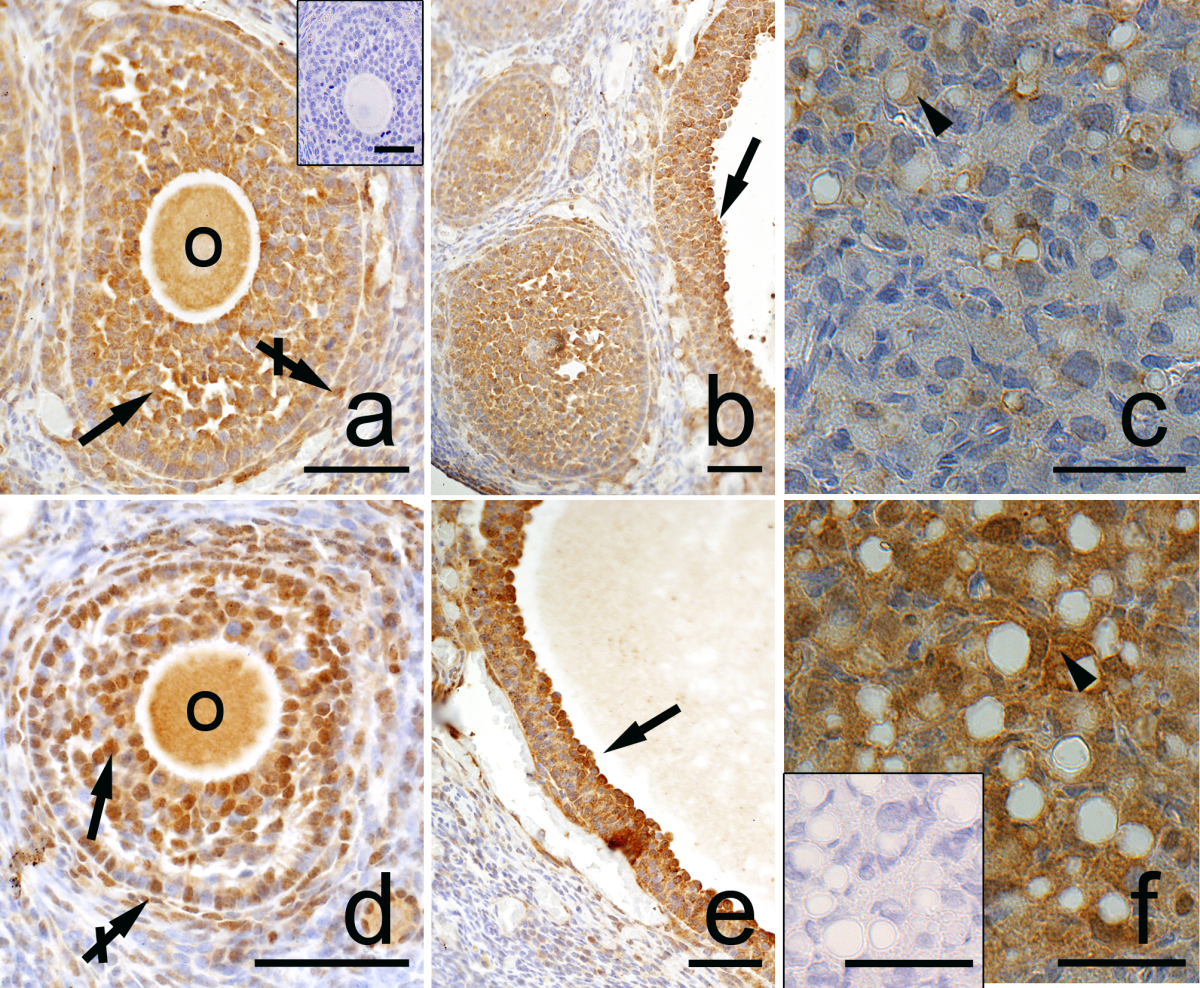
**Immunolocalization of Beclin1 in the postpartum spiny mouse ovary from the control (a, b, c) and the DEX-treated females (d, e, f).** Beclin1 is present in the oocytes (O), granulosa (arrows), and theca interna (crossed arrow) layers of ovarian follicles both in the control (a, b) and the DEX-treated females (d, e). However, the luteal cells (arrowhead) from the DEX-exposed females (f) indicated stronger staining than in the control group (c). The control sections did not exhibit any positive staining (a, f, inset). Scale bars represent: 50μm.

**Fig 9:**
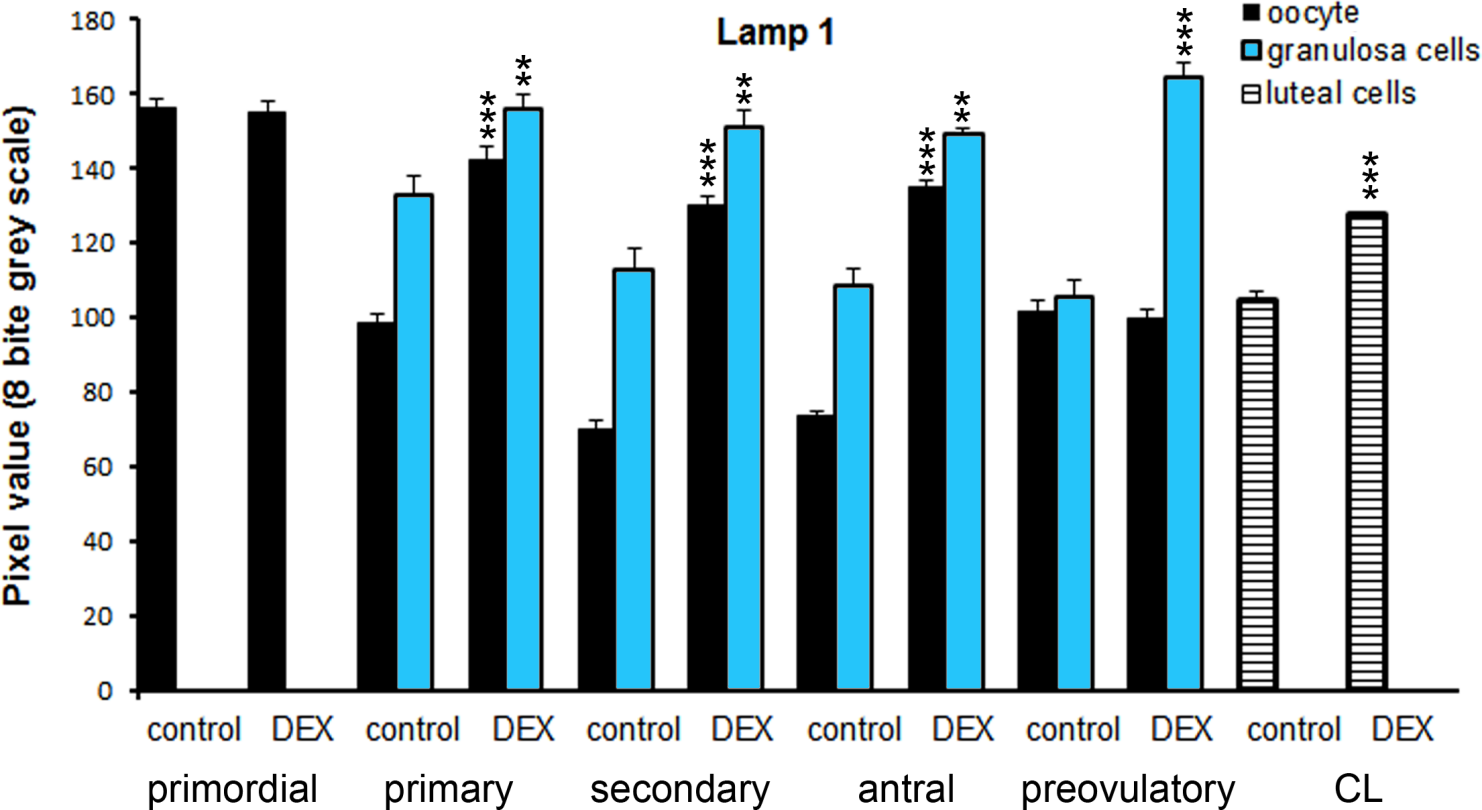
Quantitative analysis of the intensity of Lamp1 staining in ovarian follicles/corpus luteum from the control and the DEX-treated females. Data are presented as mean ± s.e.m. P-values less than 0.05 from Student’s t-test are indicated **p<0.01, ***p<0.001, CL- corpus luteum.

**Fig 10:**
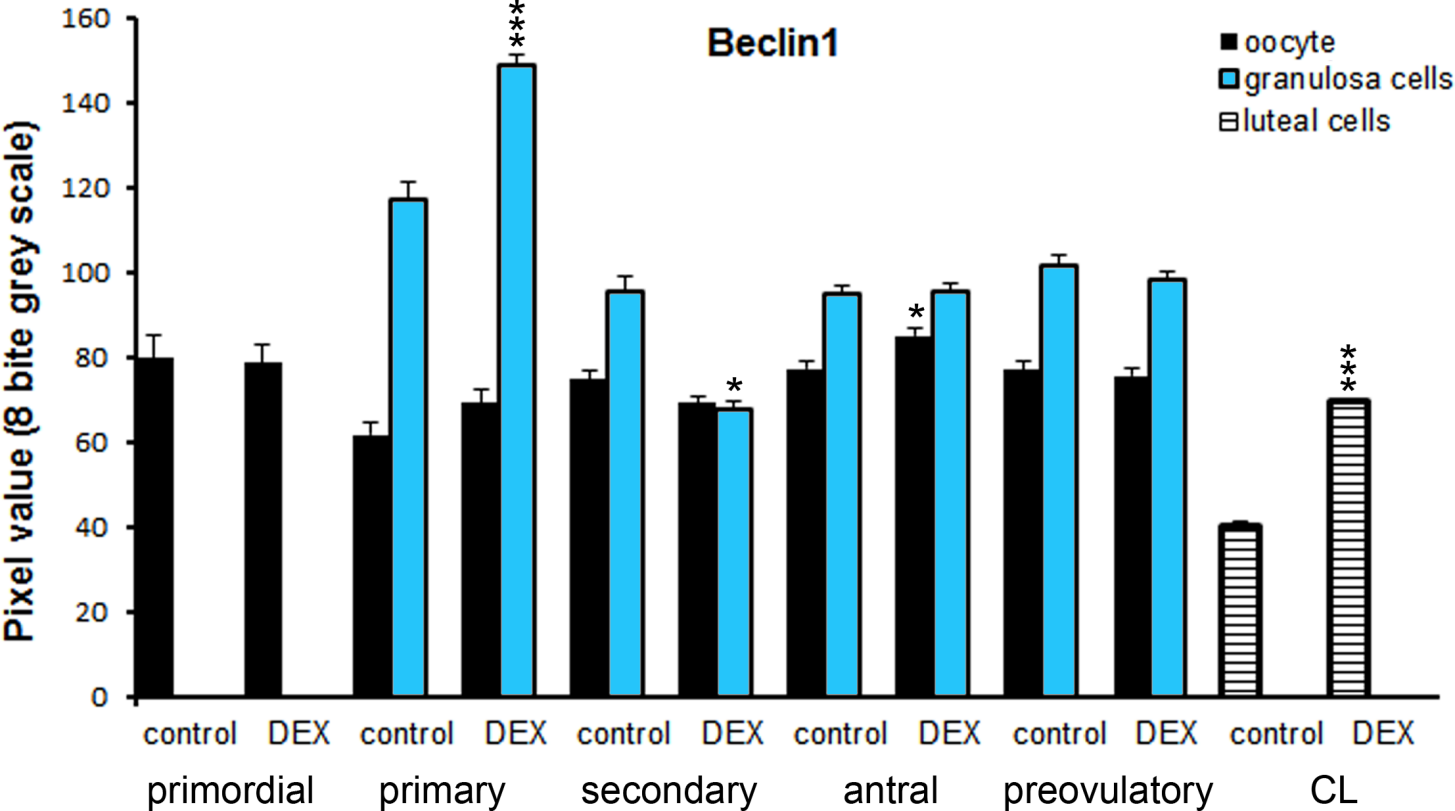
Quantitative analysis of the intensity of Beclin1 staining in ovarian follicles/corpus luteum from the control and the DEX-treated females. Data are presented as mean ± s.e.m. P-values less than 0.05 from Student’s t-test are indicated *p<0.05, ***p<0.001, CL- corpus luteum.

Both in the control and the DEX-treated group, a positive TUNEL signal was detected in the degenerating large luteal cells (Fig 3B, 3C, 3E and 3F) and granulosa layer of ovarian follicles (Fig 4). Interestingly, the apoptotic index (AI) of granulosa cells from atretic primary, secondary, antral, and preovulatory follicles as well as regressing luteal cells was significantly greater in the control females than in the DEX-treated group (Table 3). Another apoptosis marker–caspase-3 was detected in the granulosa cells and oocytes of atretic ovarian follicles as well as in the cytoplasm of large luteal cells both in the control (Fig 5A, 5B and 5C) and in the DEX-treated females (Fig 5D, 5E and 5F). In the DEX-treated group the intensity of caspase-3 staining in granulosa layer from secondary, antral and preovulatory follicles as well as in large luteal cells was significantly lower than in the control group (respectively p<0.05, p<0.05, p<0.001, and p<0.001; Fig 6). However, only oocytes from the control primordial and primary follicles indicated stronger staining in comparison with the DEX-treated group (respectively p<0.01, p<0.05; Fig 6). Immunohistochemistry revealed exclusively cytoplasmic Lamp1 as well as cytoplasmic/nuclear Beclin1 localization in oocytes, granulosa layer, and large luteal cells in both animal groups examined (Figs 7 and 8). The immunoreactivity for autophagy marker–Lamp1 was significantly stronger in granulosa layer of primary (p<0.01), secondary (p<0.01), antral (p<0.01), and preovulatory follicles (p<0.001) as well as in large luteal cells (p<0.001) from the DEX-treated group than in the control (Fig 9). In turn, the intensity of Beclin1 immunostaining in granulosa layer of antral and preovulatory follicles in DEX-treated females was comparable to the control group. Only oocytes from antral and granulosa cells from primary follicles showed stronger Beclin1 immunostaining in the DEX-treated group when compared to the control (respectively p<0.05 and p<0.001; Fig 10). Moreover, staining for Beclin1 in granulosa cells from secondary follicles was weaker than in the control (p<0.05). However, the large luteal cells from the DEX-exposed females (Fig 8F) indicated more intensive labelling than the control (Fig 8C, p<0.001; Fig 10).

**Table 3:** Effect of dexamethasone on the apoptotic index (AI) of granulosa and luteal cells in the postpartum spiny mouse ovary.

Atretic ovarian follicles	Apoptotic index (AI) %, (TUNEL assay)	
	Control	DEX-treated
primary	30.4 ± 0.9	23.6 ± 0.7 *
secondary	71.5 ± 0.8	53.3 ± 0.6 ***
antral	63.3 ± 1.4	51.0 ± 0.7 **
preovulatory	61.2 ± 1.3	50.6 ± 1.0 **
regressing luteal cells	54.8 ± 0.6	38.6 ± 0.9 ***

Values are expressed as mean ± s.e.m. P values were calculated with the Mann Whitney U-test

*p<0.05.

**p<0.01.

***p<0.001.

### GR localization

**Fig 11:**
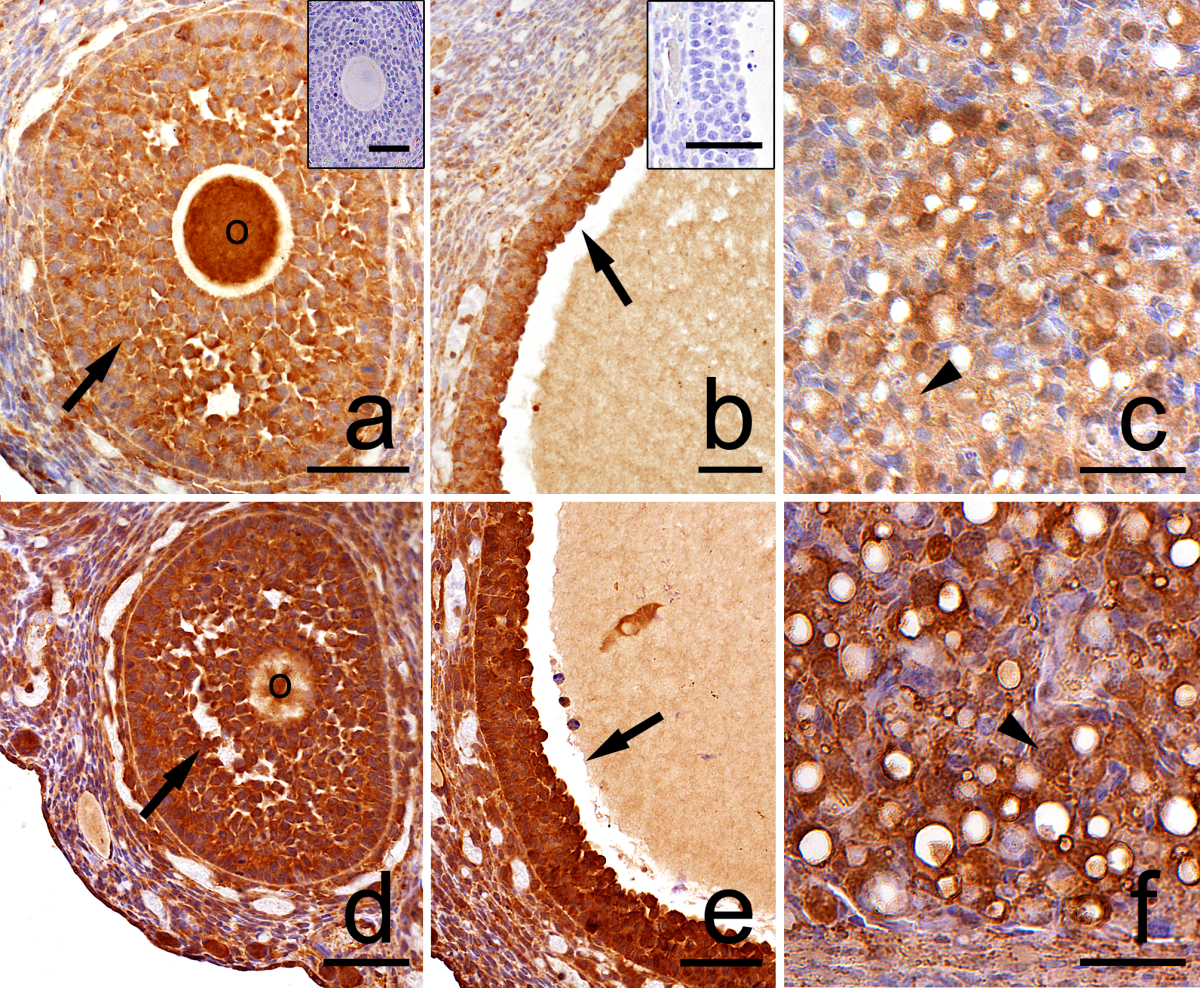
**Representative micrographs of GR immunolocalization in the postpartum spiny mouse ovary from the control (a, b, c) and the DEX-treated group (d, e, f).** In both cases, a positive signal was visible in the oocytes (O), granulosa (arrows), and luteal cells (arrowhead). However, the granulosa layer and luteal cells from the control group (a, b, c) indicated less intensive GR staining than that in the DEX-treated group (d, e, f). Control sections (a, b, inset). Scale bars: 50μm.

**Fig 12:**
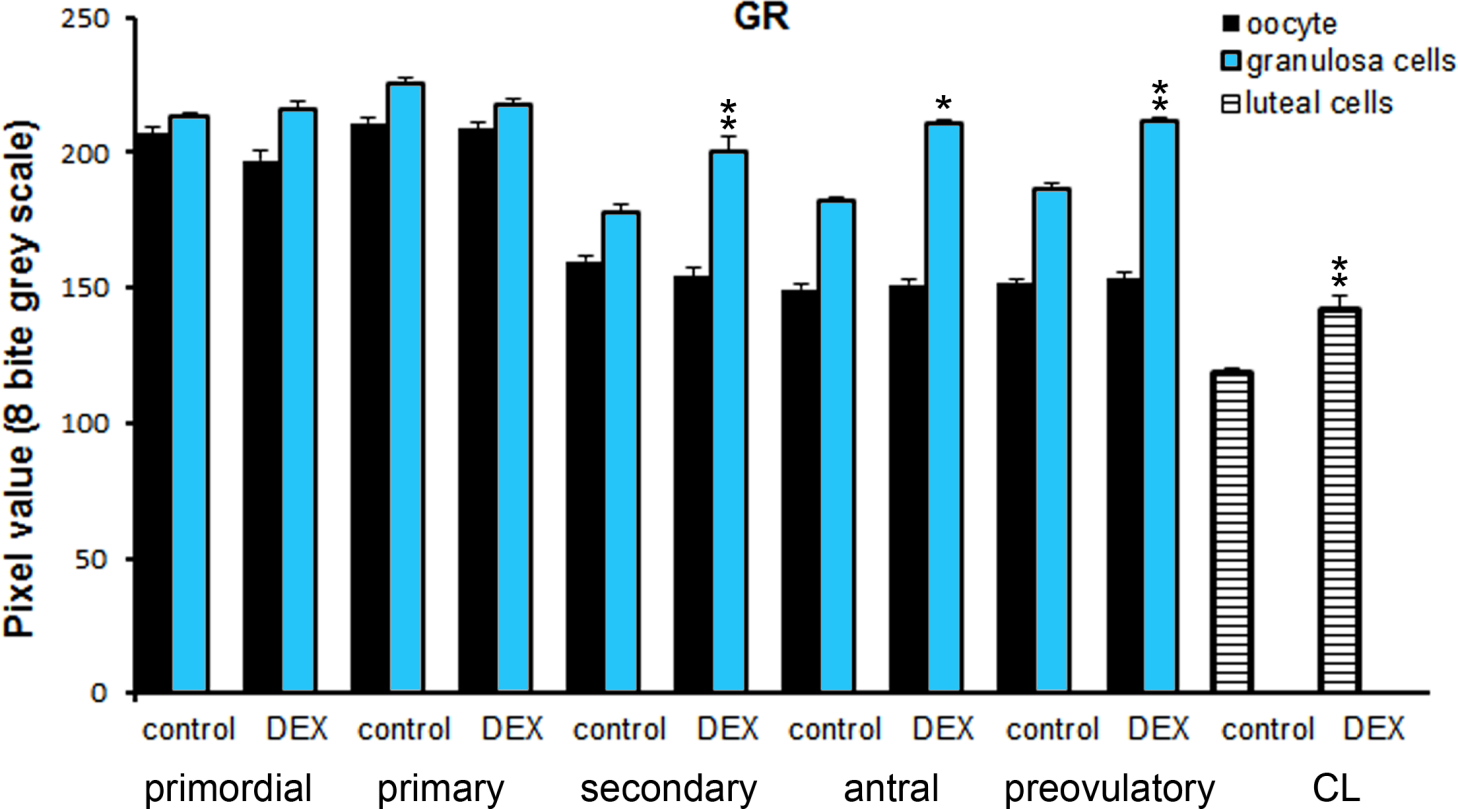
Chart represents the intensity of GR staining in ovarian follicles/corpus luteum from the control and the DEX-treated females. Data are presented as mean ± s.e.m. P-values less than 0.05 from Student’s t-test are indicated *p<0.05, **p<0.01, CL- corpus luteum.

The glucocorticoid receptor (GR) was localized in the cytoplasm of large luteal cells, in oocytes and granulosa layer of follicles as well as in stromal cells both in the control (Fig 11A, 11B and 11C) and the DEX-treated females (Fig 11D, 11E and 11F). GR immunoreactivity was significantly stronger in large luteal cells (p<0.01), granulosa layer of secondary (p<0.01), antral (p<0.05), and preovulatory follicles (p<0.01) in the DEX-treated than in the control groups. (Fig 12).

### Western-blot analysis

**Fig 13:**
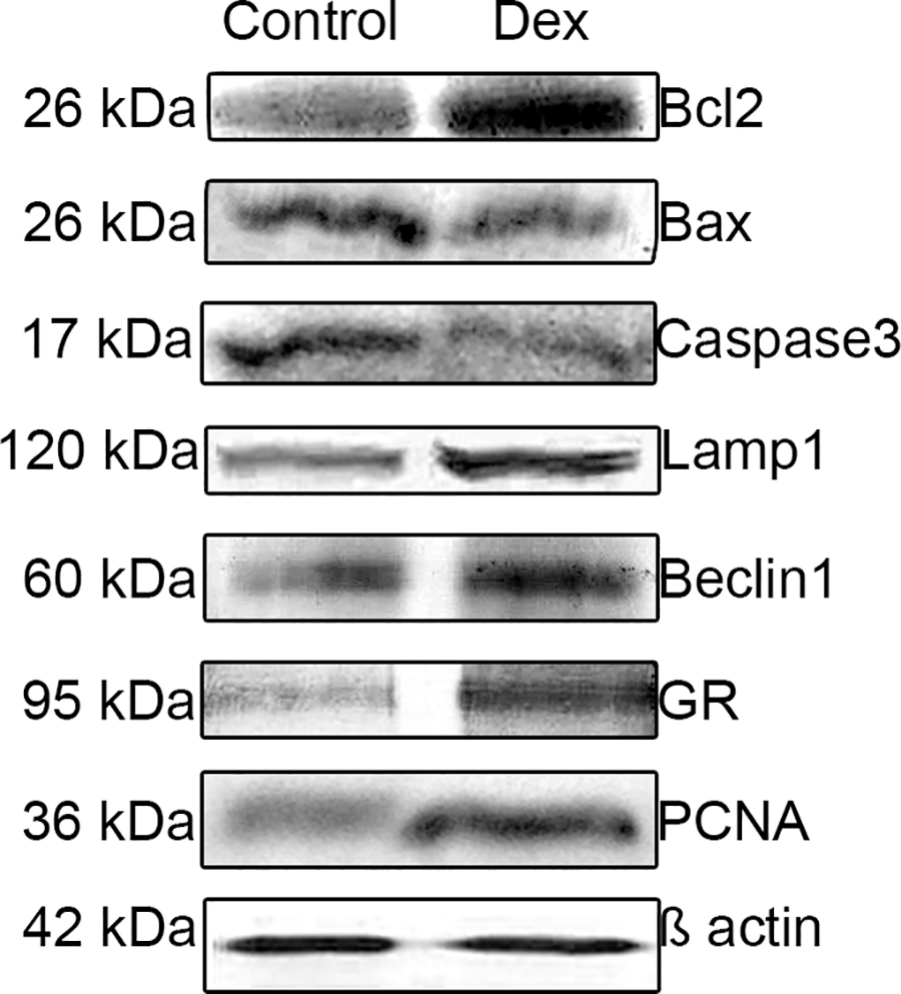
Representative Western blot of Bcl-2, Bax, caspase-3, Lamp1, Beclin1, GR and PCNA expression in the postpartum spiny mouse ovary from the control and the DEX-treated females.

**Fig 14:**
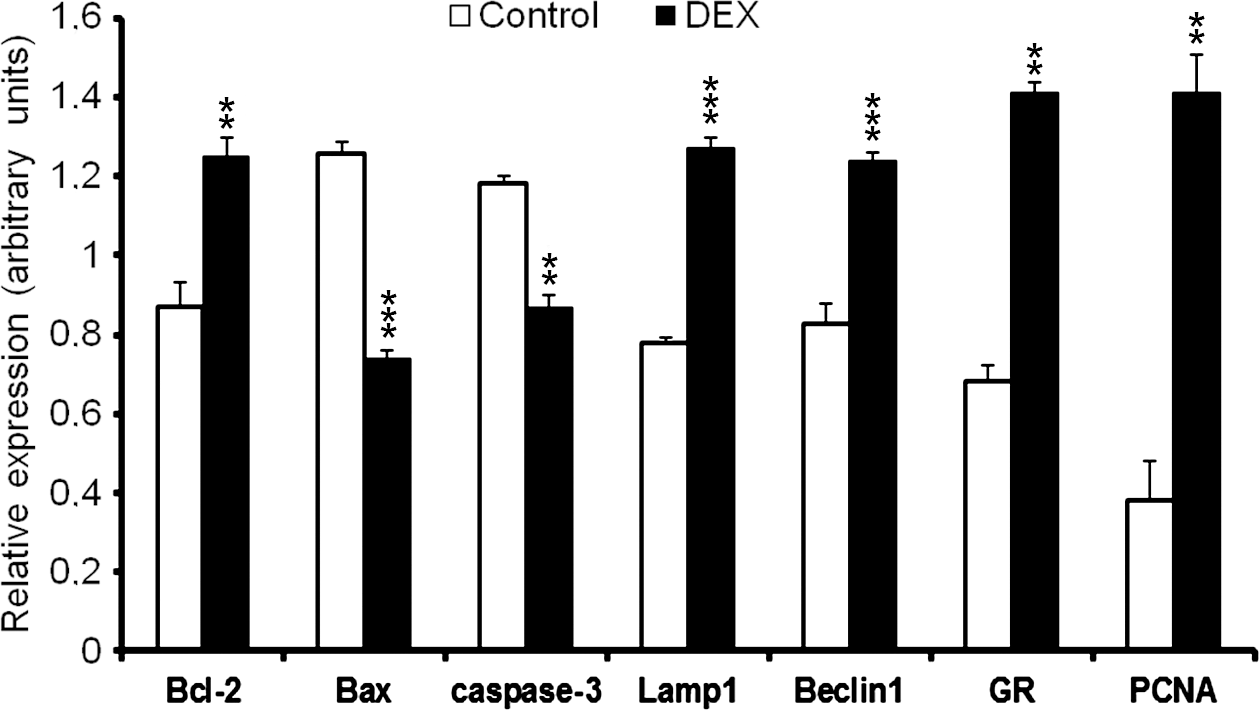
The relative expression of Bcl-2, Bax, caspase-3, Lamp1, Beclin1, GR and PCNA in postpartum ovaries obtained from the control and the DEX-treated spiny mouse. Analysis of each protein was evaluated with densitometry and expressed as the ratio relative to β-actin. Data are presented as mean ± s.e.m. P-values less than 0.05 from Student’s t-test are indicated **p<0.01, ***p<0.001.

The study revealed significantly higher expression of caspase-3 and Bax in the control group in comparison with the DEX-treated females (p<0.001; Fig 13, Fig 14). In turn, a greater expression of autophagic Lamp1 and Beclin1 proteins, Bcl-2 as well as PCNA and GR receptors were observed in the DEX-treated group (Fig 13, Fig 14). The results obtained confirmed the immunohistochemical observations.

## Discussion

The present study revealed a decreased expression of apoptosis-related (caspase-3, Bax) proteins in the postpartum spiny mouse ovary after DEX treatment. Furthermore, this study also showed that the expression of autophagy-related proteins Lamp1 and Beclin1 as well as anti-apoptotic Bcl-2 increased significantly in response to synthetic glucocorticoid. The detailed immunohistochemical studies indicated that Beclin1 and Lamp1 were strongly localized in the CL of the DEX-treated group. In addition, TEM analysis revealed the presence of numerous autophagic vacuoles as well as the accumulation of lipid droplets but maintenance of nuclear morphology in luteal cells from this group. Therefore, we propose that DEX may inhibit apoptosis and promote luteal cell autophagy in the postpartum spiny mouse ovary.

Indeed, there are many reports describing DEX as a suppressor of apoptosis for example in human gastric cancer cell line [28], bovine glomerular endothelial cells [29], human granulosa cells [30, 31], in C6 glioma cells [32], as well as in rat luteal cells [33]. There are some studies reporting that DEX can abolish apoptosis by the inhibition of prostaglandins synthesis which are the main factor involved in rat CL luteolysis [34–36]. Furthermore, Komiyama et al. [37] reported that endogenous glucocorticoid-cortisol significantly decreased apoptotic cell death by reducing caspase-3 and 8 mRNA expression and activity in bovine luteal cells. Similarly, in the present study, weak staining of caspase-3 in spiny mouse luteal cells as well as the low expression of that protein in the whole ovarian homogenate were observed in the DEX-treated females. Additionally, in the DEX-treated group the apoptotic index (AI) of luteal cells was significantly lower, (p<0.001) whereas proliferation index was significantly higher (p<0.001) when compared to the control group. It is interesting to note that differentiated, nonproliferating luteal cells show the presence of PCNA. Several previous studies also reported expression of cell proliferation markers PCNA or Ki67 during rat [38], mare [39] and cow [40] luteal regression. It is suggested that the decline in progesterone synthesis during natural CL luteolysis may promote re-entry of luteal cells into the cell cycle and susceptibility to apoptosis [40].

However, the current data show that DEX exhibits anti-apoptotic action on luteal cells and delays functional luteolysis of spiny mouse CL. Based on our finding, especially TEM analysis the participation of DEX in the induction of luteal cell autophagy and regulation of spiny mouse CL life span is suggested.

It was reported that DEX is an inductor of autophagy in lymphoblastic leukaemia cells [41] as well as in rat skeletal muscles [42], and the activation of mTOR signalling pathway is suggested. Moreover, DEX promotes lymphocytes autophagy especially when apoptosis is inhibited by Bcl-2 [43].

It is well known that the anti-apoptotic Bcl-2 protein can be involved in the induction of autophagy by interacting with Beclin1 [44]. The present study has clearly indicated that DEX can stimulate the expression of Beclin1, Lamp1 as well as Bcl-2 proteins in postpartum spiny mouse ovary. There are some data reporting that glucocorticoids, including DEX, increased the intracellular level of the anti-apoptotic protein Bcl-2 which controls cell survival rather than death [30, 31]. The expression of both Bcl-2 and Beclin1 was detected in CL during pregnancy and in persistent granulosa/luteal cells in irregularly regressing CL in climacteric women [44, 45]. These results suggest a role of autophagy in the control of cell survival. Interestingly, in the present study, the number of CLs was significantly higher (p<0.001) in the DEX-treated spiny mouse than in the control. This allows a presumption that DEX-mediated autophagy also protects the previous generation of the CL (originating from a prior pregnancy) from regression.

Furthermore, Gawriluk and Rucker [46] have demonstrated that Beclin1 and autophagy are necessary for murine CL formation, function and survival. An experiment on Bcn1 (Beclin1) knockout mice revealed impaired formation of lipid droplets in luteal cells and marked reduction in progesterone production resulting in a preterm delivery phenotype [47]. According to these authors, Beclin1 is required for accumulation of lipid droplets as well as formation and clearance of autophagosomes and endosomes. Moreover, it is necessary for murine progesterone synthesis and pregnancy maintenance. These data suggest that autophagy may play a role in steroidogenesis and thus in successful reproduction.

Furthermore, Towns et al. [48] reported marked enhancement of lipid accumulation in the CL of hypophysectomised rats associated with increased plasma 20α-dihydroprogesterone after DEX treatment. These authors suggest that DEX increased the rates of steroidogenesis. Similarly, our findings also indicated a strong accumulation of lipid droplets in the cytoplasm of spiny mouse luteal cells after DEX exposure.

On the basis of our results and available information, it can be suggested that DEX induced-autophagy represents a survival mechanism in spiny mouse luteal cells. The Beclin1/Bcl-2 complex can play an important role in the regulation of the CL life span by maintaining autophagy at levels promoting cell survival rather than cell death.

The current study demonstrated that DEX may exert its effect not only on the CL but also on follicular development. Surprisingly, in this study, the number of growing (secondary and antral) as well as preovulatory follicles was significantly higher in comparison with the control. Additionally, the proliferation index of granulosa cells in those follicle types was also increased. This finding suggests that DEX stimulates granulosa layer proliferation and promotes transition to the secondary and antral stage of follicular maturation as well as accelerates granulosa cell differentiation in the preovulatory stage. Moreover, DEX inhibits granulosa cell apoptosis. In the DEX-treated females, the number of apoptotic granulosa cells (apoptotic index) in different types of follicles was lower compared to the control. Similarly, caspase-3 staining in the granulosa layer was also weaker in the DEX-administered group. These observations are in agreement with a study by Sasson and Amsterdam [31], who reported that DEX inhibits the cleavage of caspase-3 (32kDa) and its substrate PARP (poly-ADP ribose polymerase) induced by p53 in human granulosa cells.

It was also shown that DEX stabilized the actin cytoskeleton in human granulosa cells and elevated intracellular levels of Bcl-2 [30]. Thus, this synthetic glucocorticoid may play an important role in the suppression of apoptosis in granulosa cells by enhancement of cell contact integrity, formation of actin network and upregulation of Bcl-2 expression [31, 49]. It is also reported that DEX enhanced FSH-induced P450scc (cholesterol side-chain cleavage enzyme) mRNA expression and progesterone production in pig granulosa cells [50].

Glucocorticoids exert their biological effects on cells mainly through the induction of the glucocorticoid receptor (GR). In the postpartum spiny mouse ovaries, GR receptors were observed in the cytoplasm/nuclei of oocytes and granulosa cells of ovarian follicles and in luteal and interstitial cells. Furthermore, the semi-quantitative analysis of Western-blot bands confirmed an increase in GR expression after the DEX treatment. These observations suggest that DEX can stimulate synthesis of glucocorticoid receptors, and granulosa/luteal cells are the target of its action. The local concentration of biologically active synthetic and endogenous glucocorticoids is also regulated by two glucocorticoid metabolizing enzymes 11β-hydroxysteroid dehydrogenase type1 (11βHSD1) and type 2 (11βHSD2). 11βHSD1 mainly activates the conversion cortisone to cortisol whereas 11βHSD2 inactivates cortisol to cortisone [51]. These both enzymes are developmentally regulated in human granulosa and luteal cells [52]. Non-luteinized granulosa cells abundantly express 11βHSD2 but not 11βHSD1. This reduces the level of active glucocorticoids and protects follicles from atresia (follicular apoptosis). Conversely, luteinizing granulosa cells and luteal cells possess the high level of 11βHSD1 and low 11βHSD2 which results in elevated glucocorticoids level followed by the stimulation of ovulation and CL formation [52]. DEX is a substrate for the 11βHSD2 [53] and the studies on bronchial epithelial cells indicated that prolonged treatment with DEX at therapeutic doses increased the activity of 11βHSD2 [54]. Thus, in relation to the present study it may be suggested that DEX suppresses follicular atresia partly by the inactivation of endogenous glucocorticoids. Bider et al. [55] indicated that DEX markedly decreased the level of cortisol in follicular fluid in women with polycystic ovarian disease (PCOD).

In conclusion, DEX exerts an anti-apoptotic effect on granulosa cells and stimulates granulosa layer proliferation and differentiation which accelerates follicular maturation. Furthermore, DEX induces autophagy in spiny mouse luteal cells promoting cell survival rather than cell death, which can prolong steroidogenic capacity of luteal cells and the CL life span. Taken together, DEX stimulates follicular development and prevents CL regression leading to an increase in the number of growing follicles and corpora lutea, which results in a significant increase in ovarian weight. It can be proposed from these results that DEX may maintain granulosa/luteal cell survival by a stimulatory effect on Beclin1 as well as Bcl-2 expression. Delay or overturning luteal regression by pharmacological manipulations may be of critical significance in designing strategies to improve fertility efficacy.
